# Suppression of Borna Disease Virus Replication during Its Persistent Infection Using the CRISPR/Cas13b System

**DOI:** 10.3390/ijms25063523

**Published:** 2024-03-20

**Authors:** Shigenori Sasaki, Hirohito Ogawa, Hirokazu Katoh, Tomoyuki Honda

**Affiliations:** 1 Department of Virology, Okayama University Graduate School of Medicine, Dentistry and Pharmaceutical Sciences, Okayama 700-8558, Japan; phax5815@s.okayama-u.ac.jp; 2 Department of Virology, Faculty of Medicine, Dentistry and Pharmaceutical Sciences, Okayama University, Okayama 700-8558, Japan; hogawa@okayama-u.ac.jp (H.O.); pe3w6we8@okayama-u.ac.jp (H.K.)

**Keywords:** antiviral, antivirals, Borna disease virus, CRISPR/Cas13b, persistent infection

## Abstract

Borna disease virus (BoDV-1) is a bornavirus that infects the central nervous systems of various animal species, including humans, and causes fatal encephalitis. BoDV-1 also establishes persistent infection in neuronal cells and causes neurobehavioral abnormalities. Once neuronal cells or normal neural networks are lost by BoDV-1 infection, it is difficult to regenerate damaged neural networks. Therefore, the development of efficient anti-BoDV-1 treatments is important to improve the outcomes of the infection. Recently, one of the clustered regularly interspaced short palindromic repeats (CRISPRs) and CRISPR-associated (Cas) systems, CRISPR/Cas13, has been utilized as antiviral tools. However, it is still unrevealed whether the CRISPR/Cas13 system can suppress RNA viruses in persistently infected cells. In this study, we addressed this question using persistently BoDV-1-infected cells. The CRISPR/Cas13 system targeting viral mRNAs efficiently decreased the levels of target viral mRNAs and genomic RNA (gRNA) in persistently infected cells. Furthermore, the CRISPR/Cas13 system targeting viral mRNAs also suppressed BoDV-1 infection if the system was introduced prior to the infection. Collectively, we demonstrated that the CRISPR/Cas13 system can suppress BoDV-1 in both acute and persistent infections. Our findings will open the avenue to treat prolonged infection with RNA viruses using the CRISPR/Cas13 system.

## 1. Introduction

Borna disease virus (BoDV-1) is an enveloped, nonsegmented negative-strand RNA virus belonging to the species *Orthobornavirus bornaense* [1]. BoDV-1 infects the central nervous system (CNS) of various animal species, which potentially causes various neurological diseases, such as Borna disease, a fatal encephalomyelitis in a broad range of non-reservoir mammals, e.g., horses, sheep, and alpacas [2,3,4]. Recently, human cases of fatal encephalitis caused by infections with BoDV-1 and related bornaviruses have been reported sporadically in Europe [5,6,7,8,9]. Furthermore, BoDV-1 establishes persistent infection in the CNS and potentially causes neurobehavioral abnormalities in infected animals [10,11,12,13,14,15]. Several studies have revealed BoDV-1-mediated impairments in viability and/or function of the CNS cells [11,16,17], highlighting the potential of BoDV-1 to cause CNS dysfunctions. Although the detailed molecular pathogenesis of BoDV-1-mediated impairments remains unclear, BoDV-1 infection causes CNS dysfunctions through transcriptomic changes in neuronal cells, neuronal cell losses, T lymphocyte-mediated immunopathogenesis, and/or neural network abnormalities [11,16,17,18,19,20,21,22]. Importantly, once neuronal cells or normal neural networks are lost by infection, the regeneration of damaged neural networks is poorly expected. Therefore, it is important to develop efficient anti-BoDV-1 treatments to minimize damage caused by BoDV-1 infection.

Several anti-BoDV-1 treatments have been proposed so far. Interferon-α, ribavirin, T-705 (favipiravir), and a siRNA cocktail (TD-Borna) are evaluated as antivirals against bornaviruses [23,24,25,26,27,28,29]. Among them, TD-Borna is a promising antiviral since it is effective in restricting BoDV-1 in various types of persistently infected cell lines [27]. TD-Borna is a siRNA cocktail targeting viral mRNAs encoding viral proteins, nucleoprotein (N), and the large protein (L), suggesting that viral mRNA degradation systems other than the siRNA system can be also utilized as efficient antivirals against persistent BoDV-1 infection.

Clustered regularly interspaced short palindromic repeats (CRISPRs) and CRISPR-associated (Cas) systems are the adaptive immunity of bacteria and archaea against invading foreign genetic elements [30,31,32,33]. Recently, type VI Cas effectors (Cas13) have been identified as RNA-targeting CRISPR nucleases enabling RNA degradation in mammalian and plant cells [34,35,36,37]. Among Cas13 subtypes and orthologues, the Cas13b orthologue from *Prevotella sp. P5-125* (PspCas13b) is proved to be one of the most robust and specific CRISPR nucleases for RNA degradation in mammalian cells [35]. Cas13-mediated RNA cleavage shows no protospacer flanking sequence (PFS) preference in eukaryotes, allowing for the flexible design of target regions [34,35,36,38]. By using the CRISPR/Cas13 system, several researchers have developed antiviral tools to combat various RNA viruses such as influenza A virus [39,40], lymphocytic choriomeningitis virus [39], vesicular stomatitis virus [39], severe acute respiratory syndrome coronavirus 2 (SARS-CoV-2) [40,41,42], dengue virus [43,44], hepatitis C virus (HCV) [45], and human immunodeficiency virus (HIV) [46,47]. Most of the previous studies used the CRISPR/Cas13 system for prophylaxis against these RNA viruses. On the other hand, only a few studies investigated the effect of the CRISPR/Cas13 system on established infection. For example, the CRISPR/Cas13 system was shown to suppress HIV reactivation from latently infected cells [46,47], where HIV persists as integrated DNA intermediates, but not RNA genomes. The CRISPR/Cas13 system can suppress HCV replication; however, this was demonstrated using the transient transfection of HCV replicon RNA but not a persistent infection of HCV itself [45]. Therefore, it is still unclear whether the CRISPR/Cas13 system can restrict RNA viruses in persistently infected cells.

In this study, for the first time, we evaluated whether the CRISPR/Cas13 system can suppress persistent infection with RNA viruses that have a lifecycle without any DNA intermediates. Since BoDV-1 easily establishes persistent infection without DNA intermediate, we utilized this virus as a model. BoDV-1 encodes at least six viral proteins, N, phosphoprotein (P), X, matrix (M), glycoprotein (G), and L [20,48,49,50,51,52,53,54]. BoDV-1 mainly synthesizes three mRNAs, i.e., the N, the X/P, and the M/G/L mRNAs. The M/G/L mRNA is known to be spliced, and this splicing regulates the expression of the M, G, and L genes [55,56]. Among them, we chose BoDV-1 N and M/G/L mRNAs to be targeted by the CRISPR/Cas13 system because both encode proteins, i.e., the N and the L proteins, indispensable for BoDV-1 replication. By using the CRISPR/Cas13b system targeting the N and the M/G/L mRNAs, we successfully decreased the BoDV-1 load in persistently infected cells. We also demonstrated that the CRISPR/Cas13b system targeting the N and the M/G/L mRNAs can suppress de novo BoDV-1 infection. Our results clearly indicate that the CRISPR/Cas13 system can be used as an antiviral tool to combat RNA viruses in both acute and persistent infections.

## 2. Results

### 2.1. Characterization of the CRISPR/Cas13b System in Viral mRNA Targeting in Persistently Infected Cells

**Figure 1:**
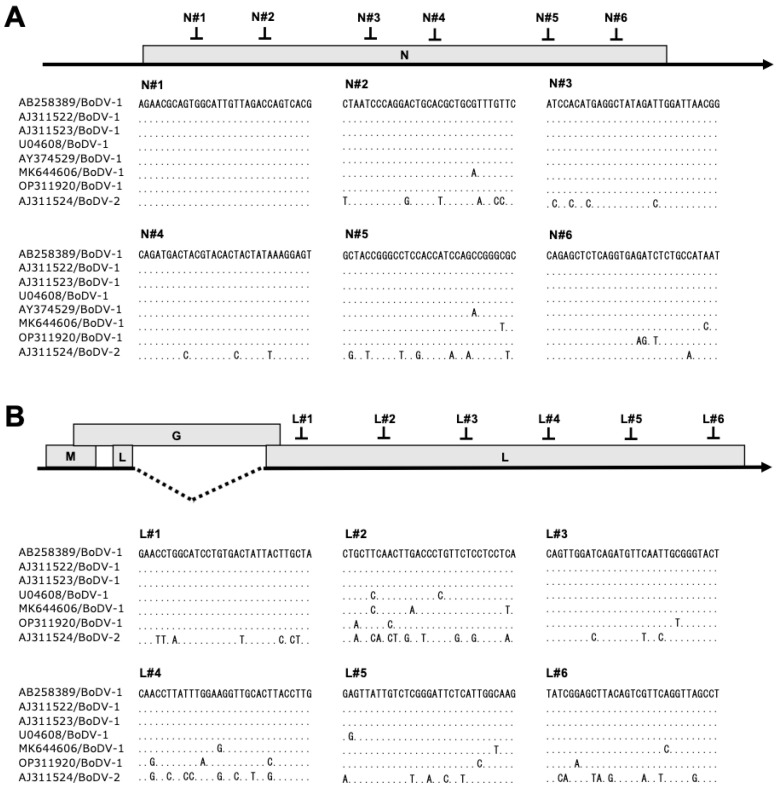
CRISPR RNAs (crRNAs) designed to target BoDV-1 mRNAs. (**A**) Schematic view of the N mRNA with the designed crRNAs (N#1–#6). Alignment of the N mRNA sequences of various BoDV-1 and BoDV-2 strains targeted by the designed crRNAs. (**B**) Schematic view of the M/G/L mRNA with the designed crRNAs (L#1–#6). Alignment of the M/G/L mRNA sequences of various BoDV-1 and BoDV-2 strains potentially targeted by the designed crRNAs. The dotted line indicates a major splicing of the M/G/L mRNA.

**Figure 2:**
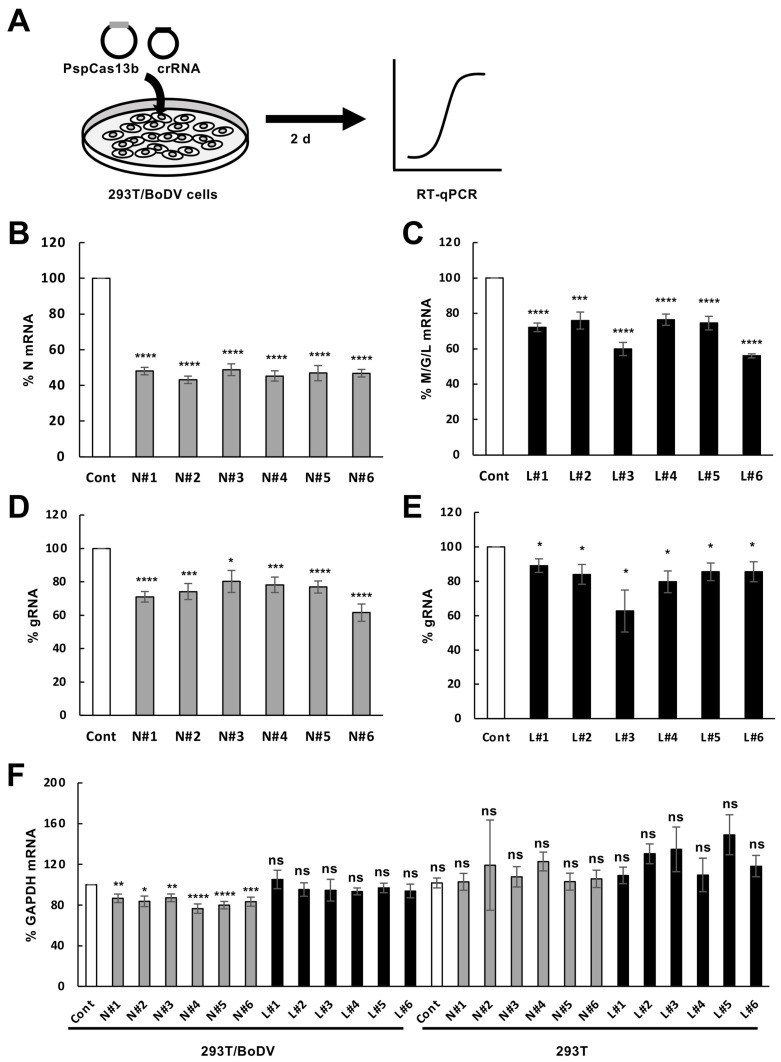
Decrease in the BoDV-1 mRNAs by the CRSISPR/Cas13b system targeting the N or the M/G/L mRNAs in persistently BoDV-1-infected cells. (**A**) Schematic representation of the protocol to evaluate the decrease in BoDV-1 RNAs using the CRISPR/Cas13b system in 293T/BoDV cells. 293T/BoDV cells were transfected with the plasmid expressing Cas13b and the indicated crRNA and then incubated for 2 days. The level of BoDV-1 RNAs in 293T/BoDV cells was determined by RT-qPCR assays. (**B**,**C**) Effects of the CRISPR/Cas13b system targeting the N (**B**) and the M/G/L (**C**) mRNAs on the level of the respective mRNAs. The levels of the N (**B**) and the M/G/L (**C**) mRNAs in 293T/BoDV cells were determined by RT-qPCR assays. (**D**,**E**) Effects of the CRISPR/Cas13b system targeting the N (**D**) and the M/G/L (**E**) mRNAs on the level of BoDV-1 gRNAs. (**F**) Collateral RNA degradation activity of Cas13b by crRNAs targeting BoDV-1 mRNAs in persistently infected cells. 293T/BoDV and uninfected 293T cells were transfected with the plasmid expressing Cas13b and the indicated crRNA and incubated for 2 days. The expression level of the GAPDH mRNA in 293T/BoDV and uninfected 293T cells expressing Cas13b and the indicated crRNA was determined by RT-qPCR assays. Values are expressed as the mean ± S.E. of at least three independent experiments. Cont, a non-target control crRNA designed in [35]; N#1–#6, crRNAs targeting different region in the N mRNA shown in Figure 1A; L#1–#6, crRNAs targeting different region in the M/G/L mRNA shown in Figure 1B. ns: not significant, *, *p* < 0.05; **, *p* < 0.01; ***, *p* < 0.005; ****, *p* < 0.001, (vs. Cont in 293T/BoDV; a two-tailed Student’s *t*-test).

We aimed to explore whether the CRISPR/Cas13b system could suppress RNA virus replication in persistently infected cells. To achieve this aim, we evaluated BoDV-1 replication in cells persistently infected with BoDV-1. We selected the N and the M/G/L mRNAs as a target because their products are indispensable for BoDV-1 RNA synthesis [49]. Although the P protein encoded in the X/P mRNA is also indispensable, targeting the X/P mRNA downregulates not only the P protein but also the X protein, a negative regulator of viral RNA synthesis. Because downregulation of the X protein may upregulate viral RNA synthesis [49], it is difficult to predict the effect of downregulation of the X/P mRNA on BoDV-1 replication; therefore, we did not target the X/P mRNA in this study. We designed six CRISPR RNAs (crRNAs) targeting the various regions in the N or the M/G/L mRNA, which are relatively conserved among commonly used BoDV-1 strains including the huP2Br (accession: AB258389) and the He/80 (accession: AJ311522) strains (Figure 1). The sequence alignment revealed that some crRNAs likely targeted diverse BoDV-1 isolates (accessions: AY374529; MK644606; OP311920) in addition to the abovementioned strains (Figure 1). To investigate whether CRISPR/Cas13b targeting using these crRNAs could directly reduce their respective target mRNAs, 293T cells persistently infected with BoDV-1 (293T/BoDV cells) were co-transfected with plasmids encoding Cas13b and individual crRNA (Figure 2A). At 2 days post-transfection, we measured the level of the N and the M/G/L mRNAs using RT-qPCR (Figure 2A). The expression level of the N mRNA was decreased to approximately 40%, with all six crRNAs designed to target the N mRNA (Figure 2B). The expression level of the M/G/L mRNA was also decreased to 60–80% with all six crRNAs designed to target the M/G/L mRNA (Figure 2C).

We then examined whether the CRISPR/Cas13b system targeting the N or the M/G/L mRNAs downregulates viral load in persistently infected cells. Because the N and the L proteins are essential for viral RNA synthesis, we reasoned that downregulation of the N or the M/G/L mRNA represses viral transcription and replication in persistently infected cells. Consistent with this hypothesis, the CRISPR/Cas13b system targeting the N or the M/G/L mRNAs decreased the level of viral gRNA to 60–90% in 293T/BoDV cells (Figure 2D,E).

In bacteria, Cas13 exhibits a nonspecific cleavage of transcripts near target mRNAs [34,57]. This kind of collateral RNA degradation activity is also reported in several mammalian cells, including 293T cells [42,58,59,60,61,62,63,64,65,66]. The collateral activity of Cas13 could activate programmed cell death in bacteria [34,57]. We therefore investigated whether the collateral activity of Cas13b was induced by the CRISPR/Cas13b system targeting viral mRNAs in persistently infected cells. To this aim, we measured the level of the GAPDH mRNA, which is unrelated to the target viral mRNA. The level of the GAPDH mRNA was slightly decreased with crRNAs targeting the N mRNAs in 293T/BoDV cells, whereas the decrease in the level of the GAPDH mRNA was not observed with crRNAs targeting the M/G/L mRNAs (Figure 2F). In addition, the decrease in the level of the GAPDH mRNA was also not observed with any crRNAs in uninfected 293T cells (Figure 2F), suggesting that activated Cas13b/crRNA complexes are required for collateral activity. Taken together, the CRISPR/Cas13b system using crRNAs targeting viral mRNAs can decrease target viral mRNAs with slight collateral activity in persistently infected cells.

### 2.2. Characterization of the CRISPR/Cas13b System Using Multiple crRNAs in Viral mRNA Targeting in Persistently Infected Cells

**Figure 3:**
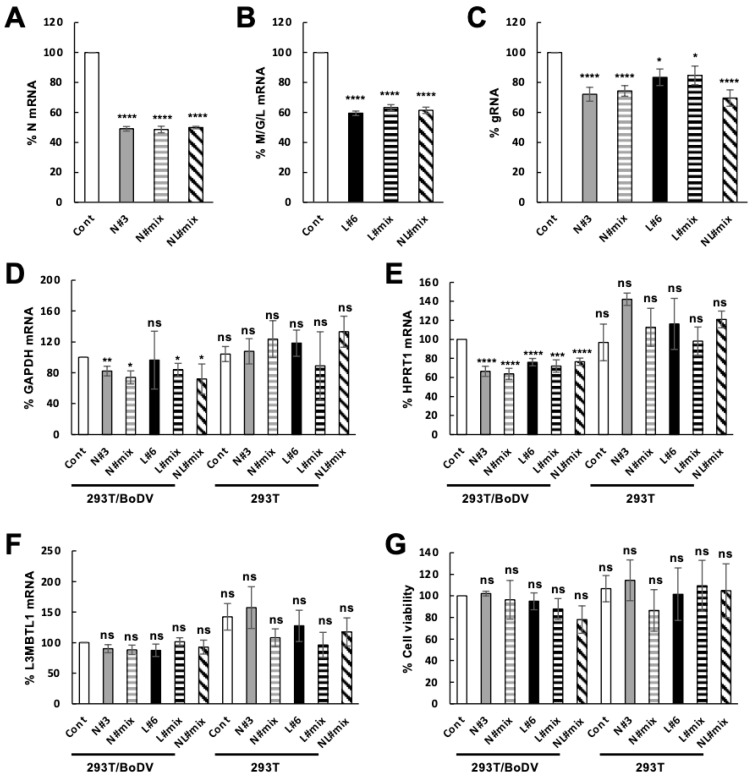
Decrease in the BoDV-1 load by the CRISPR/Cas13b system targeting the N and/or the M/G/L mRNAs in persistently BoDV-1-infected cells. (**A**,**B**) Effects of the CRISPR/Cas13b system using a combination of multiple crRNAs targeting BoDV-1 mRNAs on the level of BoDV-1 mRNAs. 293T/BoDV cells were transfected with the plasmid expressing Cas13b and the indicated single or multiple crRNAs and incubated for 2 days. The levels of the N (**A**) and the M/G/L (**B**) mRNAs in 293T/BoDV cells were determined by RT-qPCR assays. (**C**) Effects of the CRISPR/Cas13b system using a combination of multiple crRNAs targeting BoDV-1 mRNAs on the BoDV-1 load. The level of BoDV-1 gRNA in 293T/BoDV cells was determined by RT-qPCR assays. (**D**–**F**) Collateral RNA degradation activity of Cas13b by a combination of multiple crRNAs targeting BoDV-1 mRNAs in persistently infected cells. 293T/BoDV and uninfected 293T cells were transfected with the plasmid expressing Cas13b and the indicated crRNA and incubated for 2 days. The expression level of the GAPDH (**D**), the HPRT1 (**E**), and the L3MBTL1 (**F**) mRNAs in 293T/BoDV and uninfected 293T cells expressing Cas13b and the indicated crRNA was determined by RT-qPCR assays. (**G**) Cell viability of 293T/BoDV and uninfected 293T cells expressing Cas13b and the indicated crRNA. Values are expressed as the mean ± S.E. of at least three independent experiments. Cont, a control crRNA designed in [35]; N#3, a crRNA targeting the N mRNA shown in Figure 1A; L#6, a crRNA targeting the M/G/L mRNA shown in Figure 1B; N#mix, crRNAs targeting the N mRNA (a combination of crRNA N#1–N#6 in Figure 1A); L#mix, crRNAs targeting the M/G/L mRNA (a combination of crRNA L#1–L#6 in Figure 1B); NL#mix, crRNAs targeting both the N and the M/G/L mRNAs (a combination of crRNA N#1–N#6 and L#1–L#6 in Figure 1). ns: not significant, *, *p* < 0.05; **, *p* < 0.01; ***, *p* < 0.005; ****, *p* < 0.001 (vs. Cont in 293T/BoDV; a two-tailed Student’s *t*-test).

We next examined whether a combination of multiple crRNAs induces a further decrease in the level of target viral mRNA in persistently infected cells. We transfected plasmids expressing Cas13b and a crRNA targeting the N mRNA (N#3 in Figure 1A), a crRNA targeting the M/G/L mRNA (L#6 in Figure 1B), crRNAs targeting the N mRNA (a combination of crRNA N#1–N#6 in Figure 1A, referred as N#mix), crRNAs targeting the M/G/L mRNA (a combination of crRNA L#1–L#6 in Figure 1B, referred as L#mix), or crRNAs targeting both the N and the M/G/L mRNAs (a combination of crRNA N#1–N#6 and L#1-L#6 in Figure 1, referred as NL#mix) into 293T/BoDV cells and evaluated the levels of the N and the M/G/L mRNAs by RT-qPCR. The CRISPR/Cas13b system targeting the N or the M/G/L mRNAs efficiently decreased the level of the respective target mRNAs (Figure 3A,B). In contrast to our expectation, the introduction of a combination of multiple crRNAs targeting the N and/or the M/G/L did not induce a further decrease in the level of the respective target mRNAs (Figure 3A,B).

### 2.3. Downregulation of Viral Load in Persistently Infected Cells by the CRISPR/Cas13b System

We then investigated whether a combination of multiple crRNAs induces a further decrease in viral load in persistently infected cells, although no accumulative effect was detected for the level of viral mRNAs (Figure 3A,B). The CRISPR/Cas13b system targeting the N and/or the M/G/L mRNAs decreased the level of viral gRNA in 293T/BoDV cells (Figure 3C). Again, the introduction of a combination of multiple crRNAs did not induce a further decrease in the level of viral load (Figure 3C). Taken together, these results indicate that the CRISPR/Cas13b system can decrease the levels of viral mRNAs and viral gRNA even in persistently infected cells, although the introduction of a combination of multiple crRNAs does not increase the efficacy of the system against BoDV-1.

Because the CRISPR/Cas13 system targeting the N or the M/G/L mRNA exhibited collateral RNA degradation activity in 293T/BoDV cells (Figure 2F), we evaluated whether a combination of multiple crRNAs induces a further decrease in the level of nontargeted mRNAs in persistently infected cells. We measured the level of three different nontargeted mRNAs, i.e., the GAPDH, the HPRT1, and the L3MBTL1 mRNAs. The level of the GAPDH mRNA was slightly decreased with tested crRNAs except for crRNA L#6 in 293T/BoDV cells but not in 293T cells (Figure 3D). The level of the HPRT1 mRNA was decreased to approximately 70% with all the tested crRNAs in 293T/BoDV cells but not in 293T cells (Figure 3E), whereas that of the L3MBTL1 mRNA was not decreased with any tested crRNA (Figure 3F). These results suggest that the efficacy of collateral RNA degradation activity varies with each untargeted mRNA. We then examined the cell viability of cells co-expressing Cas13b and crRNA. The expression of Cas13b and crRNA did not affect cell viability regardless of the used crRNA or the infection status (Figure 3G). These results indicate that the CRISPR/Cas13b system causes slight collateral activity in persistently infected 293T cells; however, the activity is not enough to cause a decrease in cell viability. Taken together, the CRISPR/Cas13b system using crRNAs targeting viral mRNAs can decrease target viral mRNAs and viral gRNA without cell toxicity in persistently infected cells, although slight collateral activity is detected, and a combination of multiple crRNAs does not induce more efficient RNA cleavage of both target and nontarget RNAs.

### 2.4. Downregulation of De Novo BoDV-1 Infection by the CRISPR/Cas13b System

**Figure 4:**
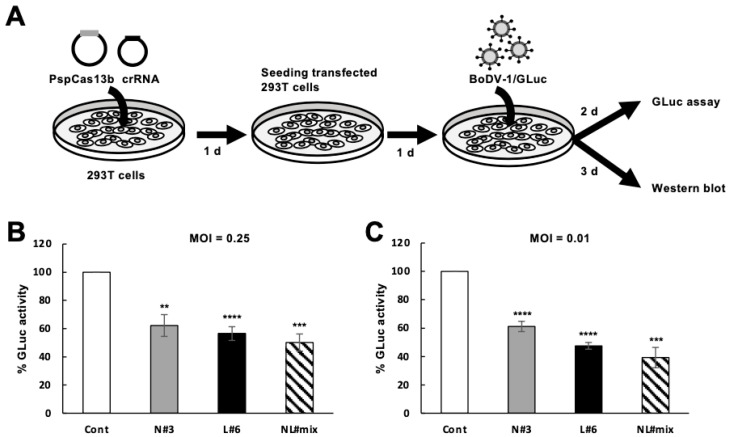
Effect of the CRISPR/Cas13b system on de novo BoDV-1 infection. (**A**) Schematic representation of the protocol to evaluate the effect of the CRISPR/Cas13b system targeting the N and/or the M/G/L mRNAs on de novo BoDV-1 infection. Uninfected 293T cells were transfected with the plasmid expressing Cas13b and the indicated crRNA. At 2 days post-transfection, the cells were infected with BoDV-1/GLuc at an MOI of 0.25 or 0.01 and further incubated for 2 and 3 days for GLuc assay and Western blot, respectively. (**B**,**C**) Effects of the CRISPR/Cas13b system targeting the N and/or the M/G/L mRNAs on de novo BoDV-1 infection. Viral infections at an MOI of 0.25 (**B**) and 0.01 (**C**) were evaluated by measuring the activity of *Gaussia* luciferase (GLuc) expressed from BoDV-1/GLuc-infected 293T cells. Cont, a control crRNA designed in [35]; N#3, a crRNA targeting the N mRNA shown in Figure 1A; L#6, a crRNA targeting the M/G/L mRNA shown in Figure 1B; NL#mix, crRNAs targeting both the N and the M/G/L mRNAs (a combination of crRNA N#1–N#6 and L#1–L#6 in Figure 1). Values are expressed as the mean ± S.E. of at least three independent experiments. **, *p* < 0.01; ***, *p* < 0.005; ****, *p* < 0.001 (vs. Cont; a two-tailed Student’s *t*-test).

We finally assessed whether the CRISPR/Cas13b system targeting the N and/or the M/G/L mRNAs suppresses de novo BoDV-1 infection. To reveal this, we first transfected plasmids expressing Cas13b and individual crRNA or combinations of multiple crRNAs in uninfected 293T cells, and subsequently, these transfected 293T cells were infected with recombinant BoDV-1 encoding the *GLuc* gene (BoDV-1/GLuc) (Figure 4A). The introduction of Cas13b together with crRNA N#3 or NL#mix decreased the amount of the N protein as expected (Supplementary Figure S1). The introduction of Cas13b together with crRNA N#3, L#6, or NL#mix decreased BoDV-1 infection at a multiplicity of infection (MOI) of 0.25 and 0.01 to approximately 50% and 40%, respectively (Figure 4B,C). However, the suppression of GLuc activity by crRNA NL#mix was comparable to that by crRNA N#3 or L#6 (Figure 4B,C). These results suggest that the CRISPR/Cas13b system targeting viral mRNAs slightly decreases de novo BoDV-1 infection.

## 3. Discussion

In this study, we demonstrated that the CRISPR/Cas13b system targeting BoDV-1 mRNAs decreases target BoDV-1 mRNAs in persistently infected 293T/BoDV cells (Figure 1 and Figure 2). We also revealed that the CRISPR/Cas13b system targeting BoDV-1 mRNAs decreases BoDV-1 load in 293T/BoDV cells (Figure 2). However, a combination of multiple crRNAs did not enhance the RNA degradation activity for both target and nontarget RNAs (Figure 3). We finally demonstrated that the CRISPR/Cas13b system targeting BoDV-1 mRNAs slightly suppressed de novo BoDV-1 infection (Figure 4). The CRISPR/Cas13 system is the first CRISPR system to enable precise RNA targeting [67]. In plants, the CRISPR/Cas13 system shows antiviral activity against plant RNA viruses such as turnip mosaic virus [68]. More recently, the CRISPR/Cas13 system has been utilized to suppress acute infection caused by several animal RNA viruses including SARS-CoV-2 [39,40,41,43,44,45,46,47]. The efficiency of viral suppression by the CRISPR/Cas13 system varies between 35–99%. To our knowledge, this is the first report demonstrating that the CRISPR/Cas13b system can reduce RNA virus replication in persistently infected cells. Although the efficiency of viral suppression was weak, our findings suggest the potential of the CRISPR/Cas13b system as an antiviral tool against persistent RNA virus infection. 

In bacteria, a double-sided PFS affects Cas13b activity [69]. On the other hand, PFS is not required for Cas13b activity in mammalian cells [35]. Although the optimal Cas13b targeting condition has not been fully revealed, the efficacy of Cas13 targeting is reportedly related to the secondary structure of the target RNAs [57]. In this study, we designed crRNA against regions relatively conserved among commonly used BoDV-1 strains (Figure 1). Some target regions, e.g., those of crRNA N#1 and L#1, were also conserved among various BoDV-1 isolates (Figure 1). Because of this, the secondary structures of these target regions likely share high similarity. The degree of sequence conservation in BoDV-1 isolates is known to be unusually high for negative-stranded RNA viruses: the maximum divergences at the nucleotide level are 3.1% and 4.1% for the N and the P genes, respectively [70]. Furthermore, the CRISPR/Cas13 system tolerates at least one mismatch between target RNA and crRNA [57]. Collectively, although evaluation by testing with a broad range of BoDV-1 isolates is required, this system could provide an antiviral tool against the infection of broad BoDV-1 isolates if crRNAs are properly designed. 

In this study, we successfully decreased BoDV-1 gRNA in persistently infected cells using crRNAs designed to target the N or the M/G/L mRNA but not viral negative-sense gRNA. Although positive-sense BoDV-1 antigenomic RNA, which is localized in the nucleus, may have been potentially targeted by our CRISPR/Cas13b system, it is unlikely because our Cas13b protein contained a nuclear export signal and was localized in the cytoplasm. The BoDV-1 replication/transcription unit is the viral ribonucleoprotein complex (RNP) that consists of the N, the P, and the L proteins together with the viral negative-sense gRNA [3,48,49]. Upon infection of negative-sense RNA viruses, the synthesis of viral proteins precedes viral replication since no progeny viral RNPs are formed without newly synthesized RNP component proteins. The CRISPR/Cas13b system targeting the N mRNA decreased the N protein (Supplementary Figure S1) and possibly the system targeting the M/G/L mRNA also decreased the L protein. Because both proteins are essential for the formation of viral RNPs, the CRISPR/Cas13b system targeting viral mRNAs suppressed the formation of active viral replication machinery and thereby reduced viral gRNA in persistently infected cells (Figure 3C and Figure 4). A similar strategy using the CRISPR/Cas13b system targeting viral mRNAs can be applied to suppress both acute and persistent infection of other negative-sense RNA viruses. 

We did not detect an accumulative antiviral effect of the CRISPR/Cas13b system by targeting BoDV-1 mRNAs with multiple crRNAs in persistent BoDV-1 infection (Figure 3). This is consistent with previous reports detecting no accumulative effect of the CRISPR/Cas13 system using multiple crRNAs [67]. The CRISPR/Cas13b system targeting the N and/or the M/G/L mRNAs also slightly suppressed de novo BoDV-1 infection; however, again, the accumulative effect was not detected (Figure 4). Once target mRNAs are cleaved by the CRISPR/Cs13b system at one site, it is enough to trigger further RNA degradation of the cleaved mRNAs by host exonucleases; therefore, multiple cleavage sites on a target mRNA were thought to have no effect on the degradation efficacy of the target mRNA. Because the N and the L proteins are essential for viral RNA synthesis, RNA degradation of the N or the M/G/L mRNA is enough to downregulate viral replication. Therefore, it was reasonable that simultaneous RNA degradation of the N and the M/G/L mRNAs did not further suppress viral gRNA. Although an accumulative antiviral effect of the CRISPR/Cas13b system by targeting BoDV-1 mRNAs with multiple crRNAs was not detected, it is still recommended to utilize multiple crRNAs against each target mRNA because of the fast evolutional rates of RNA viruses. Thus, to avoid the emergence of escape mutant viruses, targeting multiple regions in target mRNA is important. Consistently, we have demonstrated that a combination of multiple siRNAs against the N and the M/G/L mRNAs prevents the emergence of escape BoDV-1 mutants [27]. To achieve the simultaneous expression of multiple crRNAs, the development of efficient delivering systems for Cas13b and crRNAs, e.g., the all-in-one platform established in the previous report [67], is awaited. 

Cas13 exhibits nonspecific cleavage of transcripts near the target RNAs and triggers programmed cell death to limit bacteriophage infections [34,57]. Several studies have reported that collateral RNA degradation activity is not present in mammalian and plant cells [34,35,36], whereas other studies have detected collateral RNA degradation by Cas13 in some of these cells [58]. In this study, we also observed collateral RNA degradation by Cas13b in 293T/BoDV cells (Figure 2F and Figure 3D,E). However, the efficacy of collateral RNA degradation seemed not to be uniform; thus, all the tested crRNAs efficiently cleaved nontargeted HPRT1 mRNA, whereas they did not cleave nontargeted L3MBTL1 mRNA (Figure 3E,F). Although the mechanism of how collateral RNA degradation efficacy is determined is unclear, we speculate that the expression level of nontargeted mRNA, the secondary structure of nontargeted mRNA, and the amount of activated Cas13b/crRNA complexes may be important. For the GAPDH mRNA, crRNAs targeting the N mRNA exhibited collateral RNA degradation activity (Figure 2F), whereas those targeting the M/G/L mRNA did not show collateral activity (Figure 2F). Because BoDV-1 synthesized the N mRNA more than the M/G/L mRNA, target RNA-activated Cas13b/crRNA complexes were likely formed more by crRNAs targeting the N mRNA than those targeting the M/G/L mRNA. This suggests that the amount of activated Cas13b/crRNA complexes seemed to be one of the major determinants for collateral RNA degradation activity. On the other hand, a crRNA cleaved different RNAs in various degrees (Figure 3D–F), suggesting that the efficacy of the CRISPR/Cas13 system also depends on properties of nontarget mRNAs such as the secondary RNA structure and/or the expression level. Further investigation is required to understand the underlying mechanisms. 

In conclusion, we demonstrated that the CRISPR/Cas13 system targeting viral mRNAs can suppress BoDV-1 infection in persistently infected cells and a de novo infection model. Because an idea that persistent reservoirs of SARS-CoV-2 in certain tissues cause symptoms of long coronavirus disease 2019 (long COVID) has been proposed [71,72,73], any RNA viruses, including a next emerging RNA virus, may have the potential to establish persistent infection. Our findings suggest that the CRISPR/Cas13 system can offer effective therapeutics against any RNA viruses, regardless of their ability to persist in their hosts. Since we did not evaluate the effect of the system in vivo, it should be tested in the future. Because the CRISPR/Cas13 system acts in a cell-autonomous manner and the cleavage efficacy of the system is sensitive to the expression level of crRNA, highly efficient gene delivery/transduction technologies, such as lipid nanoparticles [74,75,76,77], are essential for the efficient restriction of viruses. Although the CRISPR/Cas13 system can be an attractive antiviral tool, this technical limitation needs to be overcome before practical use.

## 4. Materials and Methods

### 4.1. Cells

In this study, 293T cells (a human embryonic kidney cell line; ATCC #CRL-3216) and Vero cells (a monkey kidney cell line; ATCC #CCL-81) were cultured in Dulbecco’s modified Eagle’s medium (DMEM) (Nacalai Tesque, Kyoto, Japan), supplemented with 100 U/mL penicillin, 100 mg/mL streptomycin (Sigma-Aldrich, St. Louis, MO, USA), and 5% fetal bovine serum (FBS) at 37 °C and 5% CO_2_. Persistently BoDV-1-infected 293T cells (293T/BoDV cells) were established previously [27]. Briefly, the cells were infected with the huP2Br [78] strain of BoDV-1. BoDV-1-infected and mock-infected cells were passaged for more than 2 months exactly in the same passage history to obtain 293T/BoDV and uninfected control 293T cells. Vero cells infected with BoDV-1 expressing *Gaussia* luciferase (GLuc) were prepared as described previously [79]. 

### 4.2. Plasmids

Plasmids expressing *Prevotella sp. P5-125* (PspCas13b) and its crRNA backbone, pC0046-EF1a-PspCas13b-NES-HIV (#103862) and pC0043-PspCas13b crRNA backbone (#103854), respectively, were purchased from Addgene (Watertown, MA, USA). To generate plasmids expressing crRNA targeting the N and the M/G/L mRNAs, pairs of oligos listed in Supplementary Table S1 were annealed and inserted into the BbsI sites of the pC0043-PspCas13b crRNA backbone. 

### 4.3. Virus Preparation

The stock of recombinant BoDV-1 (based on the He/80 [80] strain) was prepared as described previously [79]. Briefly, Vero cells infected with recombinant BoDV-1 were collected and washed with phosphate-buffered saline (PBS). The cells were sonicated in DMEM supplemented with 2% FBS, and then, the samples were centrifuged at 1200× *g* at 4 °C for 20 min to remove cell debris. After centrifugation, the supernatants were collected as virus stocks and stored at −80 °C until use. 

### 4.4. Transfection

The 293T and 293T/BoDV cells were grown in 24-well plates (AGC techno glass, Shizuoka, Japan) for 24 h before transfection at 37 °C and 5% CO_2_. The cells were transfected with 210 ng of the Cas13-expressing plasmid and 280 ng of the indicated crRNA-expressing plasmids using polyethylenimine “Max” (1 mg/mL) (Polysciences, Warrington, PA, USA). Transfected cells were incubated for 2 days to evaluate the RNA levels. 

### 4.5. Virus Infection

For de novo infection, transfected 293T cells were seeded at 24 h post-transfection. At 2 days post-transfection, the cells were infected with recombinant BoDV-1 encoding the *GLuc* gene (BoDV-1/GLuc) at an MOI of 0.25 or 0.01 at 37 °C. After viral absorption for 1 h, the cells were further incubated for 2 and 3 days to prepare samples for GLuc assay and Western blot, respectively. Virus load was evaluated by GLuc activity and viral protein expression.

### 4.6. Quantitative Real-Time RT-PCR (RT-qPCR)

RT-qPCR was conducted as described previously, with some modifications [81,82,83]. Briefly, total RNA was extracted using a TRIzol reagent (Invitrogen, Carlsbad, CA, USA) according to the manufacturer’s instructions. Then, reverse-transcription was performed using a Verso cDNA Synthesis Kit (Thermo Fisher Scientific, Waltham, MA, USA) with oligo-dT primers or a huP2Br genome-specific primer (MH49). RT-qPCR assays for the GAPDH [84], the HPRT1 [85], the L3MBTL1 [59], and BoDV-1 N mRNAs were carried out using THUNDERBIRD Next SYBR (TOYOBO, Osaka, Japan) and gene-specific primers with the CFX Connect Real-Time PCR Detection System (Bio-Rad Laboratories, Hercules, CA, USA). RT-qPCR assays for BoDV-1 genomic RNA (gRNA) and BoDV-1 M/G/L mRNAs were carried out using THUNDERBIRD Next Probe qPCR Mix (TOYOBO) and the BoDV-1-specific primers and probes. The gene-specific primers and probes used in this study are shown in Supplementary Table S2.

### 4.7. Cell Viability Assay

The cell viability was determined by using a Premix WST-1 Cell Proliferation Assay System (Takara Bio, Shiga, Japan) according to the manufacturer’s instructions. Briefly, the cells were seeded at a density of 1 × 10^4^ cells/well in 96-well plates (AGC techno glass), transfected with plasmids expressing Cas13b and the indicated crRNAs, and incubated for 2 days at 37 °C and 5% CO_2_. Premix WST-1 was added to the cultural supernatant, and the cells were incubated for 3 h at 37 °C. The luminescence of cell lysates was measured using a Model 550 Microplate Reader (Bio-Rad Laboratories).

### 4.8. Western Blot

At 3 days post-infection, infected 293T cells were lysed in sodium dodecyl sulfate (SDS) sample buffer. The cell lysates were subjected to sodium dodecyl sulfate–polyacrylamide gel electrophoresis (SDS-PAGE) and transferred onto polyvinylidene difluoride membranes (Millipore, Bedford, MA, USA), which were blocked with 5% skim milk in PBS containing 0.05% Tween 20 for 1 h at room temperature, followed by incubation with mouse anti-N (HN132, [84]) and mouse anti-β-actin (Fujifilm Wako, Osaka, Japan) antibodies for 1 h at room temperature. After washing, the membranes were incubated with secondary horseradish peroxidase-conjugated goat anti-mouse IgG (H+L) antibodies (Jackson ImmunoResearch, West Grove, PA, USA). The bound antibodies were visualized with Clarity Western ECL Substrate (Bio-Rad) and detected using a MultiImager II (BioTools, Gunma, Japan). Band intensities for the N protein were measured using Image J software (ver. 1.54d bundled with 64-bit Java 8) and normalized with those of the corresponding bands of β-actin [86]. 

### 4.9. GLuc Assay

At 2 days post-infection, the GLuc activity in the culture medium was measured using a Lumat LB 9508 luminometer (Berthold, Bad WildBad, Germany) and a Pierce™ *Gaussia* Luciferase Flash Assay Kit (Thermo Fisher Scientific) according to the manufacturer’s instructions. 

### 4.10. Statistics

Statistical significance was assessed using a two-tailed Student’s *t*-test. Statistical significance was set at *p* < 0.05.

## Data Availability

The data that support the findings of this study are available from the corresponding author upon reasonable request.

## Supplementary Materials

The following supporting information can be downloaded at https://www.mdpi.com/article/10.3390/ijms25063523/s1.
