# Supplementary material for: Suppression of Borna Disease Virus Replication during Its Persistent Infection Using the CRISPR/Cas13b System
Source: Int J Mol Sci. 2024 Mar 20;25(6):3523. doi: 10.3390/ijms25063523 (PMC10971351; doi:10.3390/ijms25063523)
Supplement: Supplementary file 1 [file ijms-25-03523-s001.zip › FigS1-Cas13-revise3.pdf]

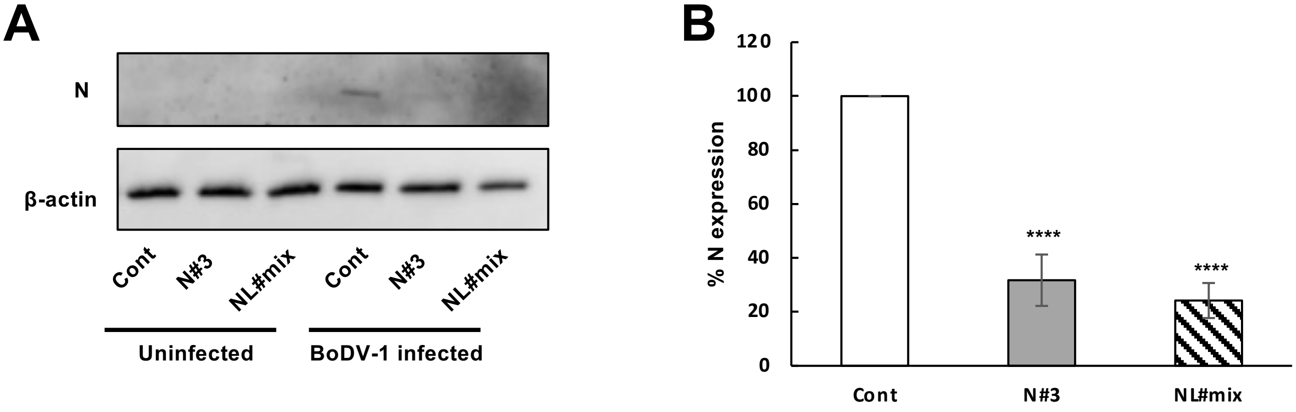

Supplementary Figure S1.

Decrease in the BoDV-1 N proteins by the CRISPR/Cas13b system targeting the N mRNAs in de novo infection. (A) The levels of the N and the β-actin proteins in BoDV-1-infected 293T cells expressing Cas13b and the indicated crRNA. Uninfected 293T cells were transfected with the plasmid expressing Cas13b and the indicated crRNA. At 2 days post-transfection, the transfected cells were seeded and infected with BoDV-1/GLuc at an MOI of 0.25. At 3 days post-infection, the cell homogenates were collected. The indicated protein levels were determined by western blot analyses. (B) Quantification of the levels of the N protein in panel (A). The band intensity of the N protein in each sample was normalized with that of β-actin. Values are expressed as the mean ± S.E. of at least three independent experiments. ****, $P < 0.001$ (vs. Cont, a two-tailed Student's *t*-test).
