# Supplementary material for: Suppression of Borna Disease Virus Replication during Its Persistent Infection Using the CRISPR/Cas13b System
Source: Int J Mol Sci. 2024 Mar 20;25(6):3523. doi: 10.3390/ijms25063523 (PMC10971351; doi:10.3390/ijms25063523)
Supplement: Supplementary file 1 [file ijms-25-03523-s001.zip › TableS1-2-Cas13(4).pdf]

Table S1. List of crRNA primers used in this study.

Primer	Sequence 5' to 3'	Description
crRNA NT forward primer (#446)	CACCGCAGGGTTTTCCCAGTCACGACGTTGTAAAA	Target Control (Cox, D.B.T. *et al.*, 2017) [35]
crRNA NT reverse primer (#447)	CAACTTTTACAACGTCGTGACTGGGAAAACCCTGC	Target Control (Cox D.B.T. *et al.*, 2017) [35]
crRNA N#1 forward primer (#529)	CACCCGTGACTGGTCTAACAATGCCACTGCGTTCT	Target BoDV-1 N mRNA
crRNA N#1 reverse primer (#530)	CAACAGAACGCAGTGGCATTGTTAGACCAGTCACG	Target BoDV-1 N mRNA
crRNA N#2 forward primer (#531)	CACCGAACAAACGCAGCGTGCAGTCCTGGGATTAG	Target BoDV-1 N mRNA
crRNA N#2 reverse primer (#532)	CAACCTAATCCCAGGACTGCACGCTGCGTTTGTTC	Target BoDV-1 N mRNA
crRNA N#3 forward primer (#448)	CACCCCGTTAATCCAATCTATAGCCTCATGTGGAT	Target BoDV-1 N mRNA
crRNA N#3 reverse primer (#449)	CAACATCCACATGAGGCTATAGATTGGATTAACGG	Target BoDV-1 N mRNA
crRNA N#4 forward primer (#533)	CACCACTCCTTTATAGTAGTGTACGTAGTCATCTG	Target BoDV-1 N mRNA
crRNA N#4 reverse primer (#534)	CAACCAGATGACTACGTACACTACTATAAAGGAGT	Target BoDV-1 N mRNA
crRNA N#5 forward primer (#450)	CACCGCGCCCGGCTGGATGGTGGAGGCCCGGTAGC	Target BoDV-1 N mRNA
crRNA N#5	CAACGCTACCGGGCCTCCACCATCCAGCCGGGC	Target BoDV-1 N

reverse primer (#451)	GC	mRNA
crRNA N#6 forward primer (#535)	CACCATTATGGCAGAGATCTCACCTGAGAGCTCTG	Target BoDV-1 N mRNA
crRNA N#6 reverse primer (#536)	CAACCAGAGCTCTCAGGTGAGATCTCTGCCATAAT	Target BoDV-1 N mRNA
crRNA L#1 forward primer (#569)	CACCTAGCAAGTAATAGTCACAGGATGCCAGGTTC	Target BoDV-1 M/G/L mRNA
crRNA L#1 reverse primer (#570)	CAACGAACCTGGCATCCTGTGACTATTACTTGCTA	Target BoDV-1 M/G/L mRNA
crRNA L#2 forward primer (#470)	CACCTGAGGAGGAGAACAGGGTCAAGTTGAAGCAG	Target BoDV-1 M/G/L mRNA
crRNA L#2 reverse primer (#471)	CAACCTGCTTCAACTTGACCCTGTTCTCCTCCTCA	Target BoDV-1 M/G/L mRNA
crRNA L#3 forward primer (#571)	CACCAGTACCCGCAATTGAACATCTGATCCAACTG	Target BoDV-1 M/G/L mRNA
crRNA L#3 reverse primer (#572)	CAACCAGTTGGATCAGATGTTCAATTGCGGGTACT	Target BoDV-1 M/G/L mRNA
crRNA L#4 forward primer (#573)	CACCCAAGGTAAGTGCAACCTTCCAAATAAGGTTG	Target BoDV-1 M/G/L mRNA
crRNA L#4 reverse primer (#574)	CAACCAACCTTATTTGGAAGGTTGCACTTACCTTG	Target BoDV-1 M/G/L mRNA
crRNA L#5 forward primer (#575)	CACCCTTGCCAATGAGAATCCCGAGACAATAACTC	Target BoDV-1 M/G/L mRNA
crRNA L#5	CAACGAGTTATTGTCTCGGGATTCTCATTGGCA	Target BoDV-1

reverse primer (#576)	AG	M/G/L mRNA
crRNA L#6 forward primer (#577)	CACCAGGCTAACCTGAACGACTGTAAGCTCCGATA	Target BoDV-1 M/G/L mRNA
crRNA L#6 reverse primer (#578)	CAACTATCGGAGCTTACAGTCGTTCAGGTTAGCCT	Target BoDV-1 M/G/L mRNA

Table S2. List of primers and probes used in this study.

Primer	Sequence 5' to 3'	Description
BoDV-1 gRNA probe (p24)	FAM-AGAACCCCTCCATGATCTCAGACCCAGA-TAMRA	Real-time RT-PCR (Hayashi, Y. *et al.*, 2009) [83]
BoDV-1 gRNA-specific RT primer for huP2Br strain (MH49)	TGTTGCGCTAACAACAAACCAATCAC	Real-time RT-PCR (Hayashi, Y. *et al.*, 2009) [83]
BoDV-1 gRNA-forward primer for huP2Br strain (OU91)	ATGCATTGACCCAACCAGTC	Real-time RT-PCR (Teng, D *et al.*, 2019) [27]
BoDV-1 gRNA-reverse primer for huP2Br strain (OU92)	ATCATTCGACAGCTGCTCCCTTC	Real-time RT-PCR (Teng, D *et al.*, 2019) [27]
BoDV-1 N-forward primer (OU277)	GGAGCCGAGCAGATCAAGAA	Real-time RT-PCR (Teng, D *et al.*, 2019) [27]
BoDV-1 N-reverse primer (OU278)	CACAAAGGAGCCTACCCAGG	Real-time RT-PCR (Teng, D *et al.*, 2019) [27]
BoDV-1 L probe	FAM-CGAGGCATCCGTGGTCAGCAGAT-TAMRA	Real-time RT-PCR (Teng, D *et al.*, 2019) [27]
BoDV-1 L-forward primer for huP2Br strain	GGAAGCGCCCCGTGTT	Real-time RT-PCR (Teng, D *et al.*, 2019) [27]
BoDV-1 L-reverse primer for huP2Br strain	CCCCCACAGTGATTCGCTTA	Real-time RT-PCR (Teng, D *et al.*, 2019) [27]
Human GAPDH-forward primer (OU67)	AGCGAGATCCCTCCAAAATC	Real-time RT-PCR (Kojima, S. *et al.*, 2014) [84]

Human GAPDH-reverse primer (OU68)	AAATGAGCCCCAGCCTTCTC	Real-time RT-PCR (Kojima, S. *et al.*, 2014) [84]
HPRT1- forward primer (HPSF-F: OU383)	GGACTAATTATGGACAGGACTG	Real-time RT-PCR (Honda, T. *et al.*, 2020) [85]
HPRT1- reverse primer (HPSF-R: OU384)	GCTCTTCAGTCTGATAAAATCTAC	Real-time RT-PCR (Honda, T. *et al.*, 2020) [85]
L3MBTL1-forward primer (#1058)	AGAGGGACAACCCACTGCTA	Real-time RT-PCR (Wang, Q. *et al.*, 2019) [59]
L3MBTL1-reverse primer (#1059)	GGCCTTCTGCTCCTCTAGGT	Real-time RT-PCR (Wang, Q. *et al.*, 2019) [59]
